# Perspectives of California Legislators on Institutional Barriers and Facilitators to Non-Partisan Research Evidence Use in State Health Policymaking

**DOI:** 10.1007/s11606-023-08547-z

**Published:** 2023-12-15

**Authors:** Neda Ashtari, Justin Abbasi, Elizabeth Barnert

**Affiliations:** 1 UCLA David Geffen School of Medicine, Los Angeles, CA 90024 USA; 2 Department of Internal Medicine, Cedars-Sinai Medical Center, Los Angeles, CA 90048 USA; 3 Department of Pediatrics, UCLA David Geffen School of Medicine, Los Angeles, CA 90024 USA

**Keywords:** policymaking, evidence-based policymaking, research translation to policy, knowledge brokers

## Abstract

**Background:**

Bridging the translational gap between research evidence and health policy in state legislatures requires understanding the institutional barriers and facilitators to non-partisan research evidence use. Previous studies have identified individual-level barriers and facilitators to research evidence use, but limited perspectives exist on institutional factors within legislatures that influence non-partisan research evidence use in health policymaking.

**Objective:**

We describe the perspectives of California state legislators and legislative staff on institutional barriers and facilitators of non-partisan research evidence use in health policymaking and explore potential solutions for enhancing use.

**Design:**

Case study design involving qualitative interviews.

**Participants:**

We interviewed 24 California state legislators, legislative office staff, and legislative research staff.

**Approach:**

Semi-structured recorded interviews were conducted in person or by phone to identify opportunities for enhancing non-partisan research evidence use within state legislatures. We conducted thematic analyses of interview transcripts to identify (1) when research evidence is used during the policymaking process, (2) barriers and facilitators operating at the institutional level, and (3) potential solutions for enhancing evidence use.

**Results:**

Institutional barriers to non-partisan research evidence use in health policymaking were grouped into three themes: institutional policies, practices, and priorities. Interviews also revealed institutional-level facilitators of research evidence use, including (1) access and capacity to engage with research evidence, and (2) perceived credibility of research evidence. The most widely supported institutional-level solution for enhancing evidence-based health policymaking in state legislatures involved establishing independent, impartial research entities to provide legislators with trusted evidence to inform decision-making.

**Conclusions:**

Potential institutional-level changes within state legislatures may enhance evidence use in health policymaking, leading to improved health outcomes and lower healthcare costs for states.

**Supplementary Information:**

The online version contains supplementary material available at 10.1007/s11606-023-08547-z.

## INTRODUCTION

Research evidence is widely underutilized in state health policymaking decisions and is increasingly ignored due to growing public distrust in science.^1–5^ A 2017 Pew research study found that most US states only modestly engage in evidence-based policymaking, often without regard for evidence quality or references to evidence during the legislative process or budget creation.^6^ An analysis of 107 “model” public health laws from 1907 to 2004 on matters such as injury prevention found that sponsors provided scientific information for only 6.5% of new laws.^7^ The evidence-policy gap is a missed opportunity to harness the power of knowledge honed through the scientific method to improve population health.^8–10^

Previous research has examined barriers operating at the individual level that contribute to the evidence-policy gap.^11^ In political science literature, individual-level barriers are characteristics of individuals that hinder research evidence use, such as policymakers’ lack of research experience or researchers’ inability to communicate research findings in a clear, useful way. Previous studies have proposed individual-level solutions, such as training researchers to communicate more effectively (“researcher push”) or teaching legislators basic research skills (“policymaker pull”).^12, 13^

Addressing individual-level barriers may facilitate knowledge transfer, but the impact and sustainability of these approaches are limited by the variability between individuals and the high rates of staff turnover in state legislatures.^14, 15^ In contrast, institutional changes target the underlying structures and processes of institutions that affect evidence-based policymaking, with potentially greater, more enduring impact. Such institutional changes require a thorough understanding of state legislators’ perspectives on institutional barriers, institutional facilitators, and institutional solutions for promoting evidence-based health policymaking. We use “institutional” to describe factors related to the structural, procedural, and cultural characteristics of legislatures that affect non-partisan research evidence use independent of any one individual.

Few peer-reviewed studies have examined legislators’ perspectives on institutional barriers, facilitators, and solutions for enhancing research evidence use in health policymaking. Existing literature also fails to differentiate between non-partisan and partisan research evidence, the latter referring to evidence provided by knowledge brokers (i.e., intermediaries who act as a bridge between research producers and end users)^16^ with political or financial conflicts of interest (e.g., lobbyists).^11–14^ Furthermore, while existing studies describe state legislator perspectives, they largely omit the views of other staff integral to the policymaking process.^17–19^ Thus, to better understand the institutional barriers that operate within state legislatures and potential institutional solutions, we examined the perspectives of California state legislators, legislative office staff, legislative research staff, and health policymaking experts.

## METHODS

We used a case study design involving semi-structured interviews with California state legislators, legislative office staff, legislative research staff, and health policymaking experts.^20^  Due to the well-documented detrimental effects of partisanship on evidence-based policymaking, we operationalized “non-partisan” as a qualifier to describe evidence provided by impartial knowledge brokers without political affiliations or vested interests in policy outcomes.^21^ For example, within legislatures, non-partisan staff typically work for the state rather than individual legislators and respond to research requests from all members of a legislature or chamber.^22^ In contrast, partisan staff are affiliated with political parties and are hired by legislators who belong to that party. They respond to research requests only from members of their own party, providing the evidence needed to develop policy positions and advance their legislative agenda. We used the term “research evidence” to describe information derived through the scientific method.^6^

The semi-structured interview guide (Appendix 1) was developed through a multiple-step process, which involved outlining the broad research questions of the study and developing probes within the major areas of barriers to research evidence use and potential solutions described in the literature. The guide queried participants’ demographics, professional roles, and perceived institutional barriers and facilitators to non-partisan research evidence use in health policymaking, and included probes tailored to respondents’ roles and areas of expertise. Interviews also explored participant attitudes toward proposed solutions identified through literature review and invited participants to share any additional solutions. The interview guide was piloted with a health professional and state legislator external to the study team.

We invited members of the California Senate and Assembly Health Committees to participate via email. To allow for greater participation, email respondents were scheduled for in-person interviews at the state capitol during summer recess, the interim period between legislative sessions during which legislators are not actively voting on bills and are relatively more available. Legislative offices that did not respond via email were approached in person and invited to participate. Participants from legislative offices included legislative aides, legislative directors, and lawmakers (i.e., senators or assemblymembers). We then used snowball sampling to expand our sample. Interviews were conducted by the lead author (NA) who had expertise in public health policy.

Participants received the interview guide at least one day in advance of the interview. The purpose of the study and content of the interview were explained to participants in detail. Interviews were audio recorded with participant permission and transcribed. Interviews lasted 30 to 45 minutes. We conducted 17 interviews in person and seven interviews via telephone. We conducted 22 interviews from July to September 2019; two additional interviews were deferred to July 2021 due to the COVID-19 pandemic. The additional interviews confirmed saturation of major themes, defined as a repetition of the same ideas without the introduction of new ideas.^23^

Two members of the study team (NA, CB) independently performed qualitative thematic analysis, per Braun and Clark (2006), to identify emerging themes about barriers, facilitators, and potential solutions for increasing non-partisan research evidence use in state health policymaking.^24^ Coders familiarized themselves with the data by closely examining transcripts and noting initial ideas (i.e., without predetermined categories). Initial codes were generated and applied to identify when and how research evidence is used throughout the legislative process, barriers and facilitators to research evidence use, and solutions to enhance evidence use as contained within the interview guide and proposed by interviewees. After refining the codebook, codes were applied to transcripts in Dedoose software 1.3.34 (SCRC, Manhattan Beach, CA). The initial codebook was then revised to develop a secondary codebook before conducting another round of coding to differentiate between individual-level and institutional-level factors, ensuring that our definitions were consistent with existing literature. Specifically, barriers and facilitators were coded as “institutional-level” if they described characteristics of the legislature that could be identified without reference to individual members (e.g., size, complexity), as compared to “individual-level,” which described characteristics of individual actors within the legislature (e.g., policymaker education level).^21, 25^ Through these iterative rounds of coding, the central themes on research evidence use emerged. A fourth round of coding identified the highest-yield strategies for enhancing evidence use by categorizing each solution based on perceived feasibility and impact. Disagreements among the coders were discussed and resolved at broader weekly research team meetings. The same two coders were involved in all rounds of coding.

We then performed member checking with an interviewee with substantial experience serving the state health committees. To further validate our results, we debriefed findings with two academic health services researchers external to the study team with expertise in state health policymaking. Our university’s institutional review board approved study procedures.

## RESULTS

In total, 24 of 26 (92%) individuals contacted accepted invitations to participate; those who declined cited scheduling conflicts. Participants included state legislators, legislative office staff, legislative research staff, and health policymaking experts. Table 1 describes participants’ roles and Appendix 2 defines each role. Participants reflected the composition of the state legislature with regard to race, ethnicity, age, gender, and level of educational attainment. All participants held college degrees, with 88% (21of 24 participants) possessing a master’s degree or higher. Of the 21 participants from legislative offices (i.e., legislative aides, legislative directors, or lawmakers), 15 represented Democratic offices and six represented Republican offices.

**Table 1:** Participant Roles

Position	Total *n* = 24
Legislator	3
Legislative office staff*	12
Legislative research staff^†^	6
Other health policymaking experts (external to legislature)^‡^	3

^*^Legislative directors and legislative aides

^†^Legislative researchers hired by legislative committees and employees of the California Health Benefits Review Program, California Research Bureau, Senate Office of Research, and Legislative Analyst Office

^‡^Think tank and policy advocacy organization leaders

**Fig. 1:**
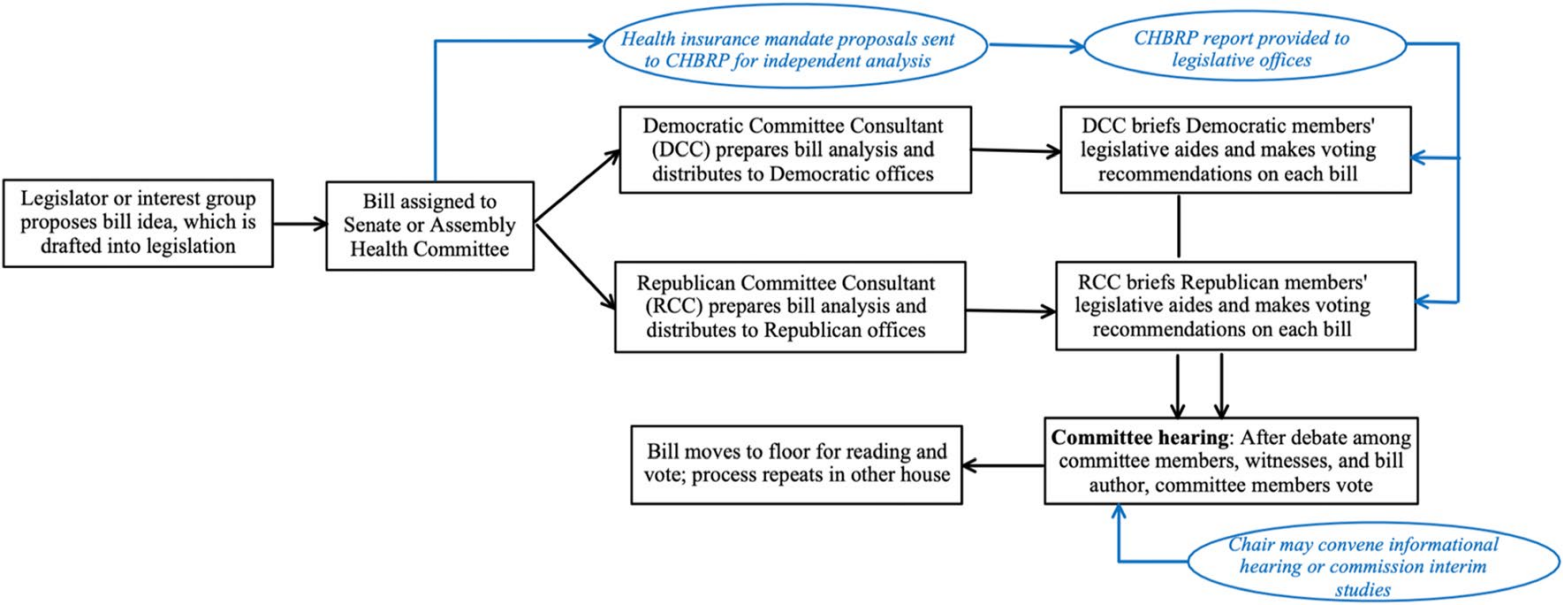
Current Research Evidence Use in California State Health Policymaking. *CHBRP*, California Health Benefits Review Program.

Interviewees felt that non-partisan research evidence was predominantly used within policy committees (Fig. 1) but significantly underutilized throughout the legislative process due to institutional barriers that limit access to and capacity for engaging with non-partisan research evidence and diminish the perceived credibility of the research evidence provided. Interviews also highlighted two entities within California that successfully enhance evidence use in health policymaking. By examining these exemplary models, characteristics of successful knowledge brokers emerged that could be more broadly incorporated into solutions to enhance evidence use across various contexts. These institutional barriers, facilitators, and solutions are discussed further below.

### Institutional Barriers

Three types of institutional barriers hindered evidence-based health policymaking: institutional policies, institutional practices, and institutional priorities of the state legislature (Table 2).

**Table 2:** Institutional Barriers to Non-partisan Research Evidence Use in State Health Policymaking

Policies	• The increasing number of bills introduced per session creates time constraints that limit non-partisan research evidence use • Inflexible legislative calendars and poorly coordinated deadlines cause unevenly distributed workloads across sessions, often leading to hasty decision-making • Term limits and low salaries increase staff turnover, diminishing institutional knowledge about non-partisan research evidence resources
Practices	• Legislative research staff are appointed by political leadership, decreasing their credibility as impartial knowledge brokers • Committee chairs select expert witnesses to testify in informational hearings, who legislators often perceive as biased • Committee chairs make voting recommendations to committee members, disincentivizing use of research evidence in legislative decision-making
Priorities	• Political calculus often outweighs research evidence, particularly for controversial issues • During periods of financial strain, disproportionate downsizing of non-partisan research staff decreases access to non-partisan research evidence • Underinvestment in academia-legislature partnerships limits access to non-partisan research evidence and creates a disconnect between legislative and academic research agendas • Declining investment in jobs and fellowships that place academic researchers in legislative positions reduces expertise and access to non-partisan research within the legislature

#### Barrier 1: Institutional Policies

Participants emphasized that the *increasing number of bills introduced per session* significantly limited policymakers’ access to and capacity for engaging with non-partisan research evidence. As one interviewee stated, “Because of the way our schedules work with deadlines, hearings have 25–30 bills at a time. How much time can you devote to all of those issues? You can’t give them the time, necessarily, that they deserve.” Time constraints caused by insufficient staffing were exacerbated by an *inflexible legislative calendar and poorly coordinated legislative and budget deadlines*, which often led to rushed decision-making and uneven workloads throughout legislative sessions. As one interviewee stated, “The way the May Revise works is—they dump thousands of pages of paper and ten new ideas on us, and the budget has to be passed by the end of this month.” Interviewees explained that, prior to bill limit increases, staff had already contended with the challenge of accessing impartial sources of research evidence (e.g., academics) in a timely fashion due to the relatively slow response times of academicians compared to lobbyists. Worsening time constraints further limited policymakers’ ability to independently review the evidence for policy proposals, increasing their reliance on lobbyists and party leadership for voting recommendations, especially during “bottleneck” periods.

Participants also shared that *term limits and low salaries* contributed to high staff turnover, which diminished institutional knowledge about non-partisan research resources (California legislators can serve up to 12 years across both chambers). One interviewee commented, “People come and go really quickly, and I assume that if you are not really interested in developing a career here, you’re not as likely to care about learning the nuances on where information comes from, and just look over whether or not it reinforces your position.”

#### Barrier 2: Institutional Practices

Institutional practices hindered non-partisan research evidence use by compromising the credibility of research evidence presented and creating power dynamics that discouraged independent thinking and robust debate about legislation. For example, interviewees explained that the political appointment of legislative research staff within committees and the inclusion of partisan policy recommendations in the bill analyses they prepared *diminished the credibility of the research evidence they provided to lawmakers. *One interviewee described how the political affiliations of research staff diminished their credibility, stating, “If you’re in the Senate, you have the Senate Office of Research. But their boss, at the end of the day, is the political leadership in the Senate. So, I think there’s an assumption among our staff that information from the Senate Office of Research and Legislative Analyst Office is going to be leftist. Just saying you’re non-partisan doesn’t make you non-partisan.” Furthermore, hiring research staff based on political affiliation rather than topic-specific expertise increased their reliance on lobbyists for research. As one research staff member explained, “I have a [social science] degree. I don’t have any scientific background … We don’t use research if we can’t really digest it, so lobbyists are really helpful.” 

Interviewees also viewed the legislature’s strong centralization of power as a barrier to evidence-based policymaking. In California, *committee chairs determine which bills are considered in their committees and control informational hearings; for example, by determining whether to hold a hearing and hand-selecting expert witnesses* who are often perceived as biased. As one interviewee remarked, “Every time Democrats would say, ‘Hey, we’re going to have an informational hearing,’ and we would offer up people for a competing view, they would just cancel the informational hearing. I don’t want to be a negative Nancy, but they tend to stack the testimony in favor of the direction that they want to go.” This centralization was also reflected in the* high prevalence of “vote whipping,” the practice of party leadership manipulating lawmakers’ voting behavior. This practice pressured members to overlook research evidence *due to the political repercussions of not voting with their party, including decreased legislative office funding.

#### Barrier 3: Institutional Priorities

Interviewees shared that *political calculus often outweighed research evidence in decision-making*, especially for controversial issues (e.g., vaccine mandates, reproductive rights). One participant explained, “Typically, high-level research gets sort of de-prioritized with other factors like questions about how this impacts our members’ districts, what is the political calculus on voting yes or no, who's in opposition—those sorts of factors play more of a role.”

Interviewees noted that the legislature’s insufficient prioritization of research evidence was demonstrated by the historically *disproportionate reduction of non-partisan research staff and the elimination of certain research agencies altogether during periods of financial strain*. One interviewee explained that, “During the budget crunch, they drastically curtailed the size of legislative staff and got rid of the Assembly Office of Research. The CRB [California Research Bureau] was created as the backstop to mitigate the loss of analytical ability in the legislature… The result was that people who were subject matter experts were the lobbyists now.”

In addition to reducing expertise internally, *historical underinvestment in formal academia-legislature partnerships* also reduced legislators’ access to external expertise. As one interviewee described, “We tend to go to our usual suspects in terms of finding information. I’ll use the UC [University of California] labor folks, and I’m sure there are other research arms of the UC that might be relevant to me, but I don’t have a mechanism to find them.” While some communication between academicians and policymakers occurred through informal relationships, interviewees emphasized that the lack of funded formal partnerships led to systematic underutilization of academic experts who could offer non-partisan research and an overreliance on lobbyists. The lack of academia-legislature partnerships also led to a perceived disconnect between many research questions and pressing policy questions. One interviewee stated, “Something I’ve found coming from science to the policy world is sometimes real-world problems have not been addressed in research or haven’t been addressed in the way that they actually manifest themselves in real society. And real society is what we’re trying to make better.”

Finally, participants identified the state’s *declining investment in jobs and fellowships designed to enhance evidence-based policymaking* as a barrier to non-partisan research evidence use. This included the discontinuation of a UC graduate student policy research program and the downsizing of a science fellowship program that placed UC doctoral students in legislative offices to provide expertise to policymakers. Underscoring the crucial role of science fellows within the legislature, one interviewee stated, “It’s more a matter of people having the expertise on how to search for literature and the expertise of being able to read the literature. You need a lot of training, particularly since almost nobody on the committee staff has a scientific background… If you don’t have a research background, I don’t see how you could read papers… And I don’t think you’re going to find things when you try to search for them unless you have a lot of experience, which is the advantage we have with the science fellows here.” While legislators could author legislation specifying that a law will go into effect only upon the completion of a university-led research study, interviewees expressed that such instances were rare given the lack of funding for government-commissioned research.

### Institutional Facilitators

In describing institutional facilitators of non-partisan research evidence use, participants frequently referenced two independent entities serving the legislature: the CRB, a branch of the state library that responds to research requests from legislative staff, and the California Health Benefits Review Program (CHBRP), a university-affiliated research agency that provides the legislature with multidisciplinary evidence summaries related to all proposed health insurance mandates.

**Fig. 2:**
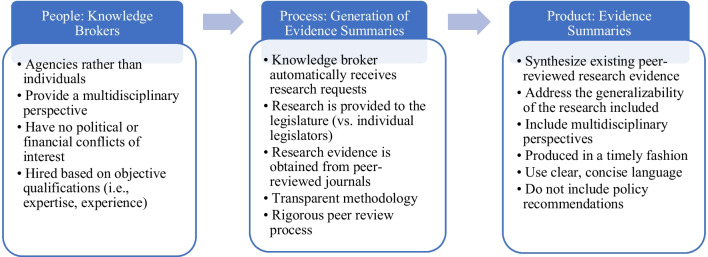
Key Characteristics of Effective Knowledge Brokers that Enhance Non-partisan Research Evidence Use in State Health Policymaking

Interviewees identified several characteristics that rendered these knowledge brokers as universally trusted, highly utilized sources of research evidence among legislators (Fig. 2). For example, both agencies are financially independent from the legislative and executive branches, preventing funding concerns from affecting the research evidence they produce. They are also strictly non-partisan: staff are neither employed nor appointed by political parties and are forbidden from disclosing their political beliefs within the workplace. To further bolster their perceived neutrality, the CRB and CHBRP refrain from including policy recommendations in their research products. Additional positive attributes of the agencies included their conflict-of-interest policies, transparent methodologies, rigorous peer review processes, and university affiliations, which allowed a broad range of experts to contribute to research analyses, providing the crucial multidisciplinary lens needed for issues as complex as health policy. Describing the legislature’s high regard for CHBRP, one interviewee stated, “Lawmakers might be disappointed one time at a finding that is not as favorable as they might have hoped, and the next time, they’re pleased. But ultimately, it’s the fact that everyone feels like it’s a neutral, rigorous source that allows them to argue the merits of a policy rather than hack at each other’s estimates. They’re arguing on an accepted baseline.”

### Institutional Solutions

Solutions that targeted researchers’ ability to disseminate research findings (“researcher push”) and policymakers’ ability to interpret them (“legislator pull”) were viewed as less impactful than solutions that involved independent, third-party knowledge brokers (e.g., CHBRP). Reasons included time constraints on policymakers and researchers, high turnover rates within legislatures, the limited power of individuals compared to agencies, and the potential for individuals to “cherry-pick” evidence. Findings regarding institutional solutions were organized into the same three categories as findings regarding institutional barriers.

#### Solution 1: Institutional Policies

While interviewees strongly supported increasing bill introduction limits, this policy change was considered infeasible because of legislators’ tendency to oppose restrictions on their legislative powers. However, increasing flexibility in the legislative calendar and revising legislative and budget deadlines were considered helpful for easing existing time constraints. Interviewees acknowledged that removing term limits could incentivize and empower staff to utilize research evidence but generally opposed this solution due to the benefits of term limits and the difficulty of amending state constitutions to remove them.

#### Solution 2: Institutional Practices

Participants held varied opinions regarding changing committee practices to improve non-partisan research evidence use. Republican interviewees supported using objective criteria to select expert witnesses and provide a more balanced range of perspectives during hearings, while Democrats raised concerns about potentially providing a platform for “pseudo-scientists” to spread misinformation. In contrast, participants unanimously supported using a third-party staffing bureau to hire legislative research staff based on subject matter expertise rather than political affiliation. Consultants would then be hired as state employees rather than employees of their legislative caucus, thereby mitigating the effects of partisanship on bill analyses. Interviewees also supported adopting conflict-of-interest policies within committees, removing policy recommendations from bill analyses, and prohibiting committee leadership from making voting recommendations to encourage legislators to independently assess research evidence to guide their decision-making.

#### Solution 3: Institutional Priorities

Participants unanimously viewed establishing and expanding independent research agencies such as CHBRP as the most impactful and feasible solution to enhance evidence use. Participants also supported investing in tools and programs that would facilitate bidirectional communication between the legislature and universities, including joint workshops and seminars, policy fellowships, policy advisory boards comprised of both legislators and university experts, and government-commissioned collaborative research projects that would address real-world policy questions. Interviewees felt that strong connections with academia would reduce the legislature’s reliance on lobbyists, with one participant stating, “It would be great if academics contacted us more—if our bill ideas were driven by evidence instead of lobbyists.” Participants explained that stronger university-legislature partnerships would also provide access to a diverse range of experts, increasing policymaker trust in the evidence provided and counteracting the influence of special interests.

## DISCUSSION

State policymaker perspectives indicate that legislators recognize the value of non-partisan research evidence but underutilize it in health policymaking due to institutional barriers related to the policies, practices, and priorities of state legislatures. Our findings and extant literature suggest that state governments can promote evidence-based health policymaking by increasing access to and capacity for engaging with non-partisan research evidence and mitigating perceived bias among institutional knowledge brokers.^21, 26^ While the identified institutional barriers largely align with prior research,^8, 21, 26^ identified institutional solutions merit further consideration.

### Promising Institutional Solutions

Although interviewees represented a single state, substantial literature indicates the challenges they described are experienced across state legislatures, suggesting potential generalizability of several proposed solutions.^26, 27^ Recommendations for enhancing evidence-based health policymaking across state legislatures are described below and in Table 3.

**Table 3:** Promising Institutional Solutions Within State Legislatures to Enhance Non-partisan Research Evidence Use in State Health Policymaking

Solution	Mechanism
Increase capacity to utilize non-partisan research evidence	Implement bill introduction limits and create a balanced bill-to-staff ratio
	Adopt “aging requirements” that ensure legislators have adequate time to read bill proposals before voting on them
	Incorporate flexibility into legislative calendars (e.g., extended sessions, pre-session planning, interim committees)
	Revise budget and legislative deadlines to distribute work evenly across sessions
	Establish expert advisory groups that convene when urgent scientific advice is needed (e.g., United Kingdom’s Scientific Advisory Group for Emergencies [SAGE] provides scientific and technical advice to the federal government during crises and emergencies, particularly when dealing with public health threats such as pandemics, disease outbreaks, and other disasters)
Invest in formal university-government partnerships	Commission university research that answers pressing public health questions for which additional evidence is needed (e.g., Washington State Institute for Public Policy carries out pragmatic research at the direction of the legislature to answer relevant policy questions)*
	Establish and expand university-affiliated non-partisan research agencies to serve as knowledge brokers by synthesizing existing research evidence (e.g., California Health Benefits Review Program, CHBRP)
	Create opportunities for knowledge exchange and relationship building between universities and legislatures (e.g., Wisconsin Family Impact Seminars present legislators with research evidence relevant to current legislation)
	Fund non-partisan science policy fellowships in the legislature for doctoral students (e.g., California Council on Science and Technology Policy Fellowship)
	Develop directory of academic experts willing to provide research evidence to legislative offices in a timely fashion (e.g., California state university system maintains a directory of academic experts who are available for legislators to consult on a range of policy issue)*
Bolster the credibility of research evidence provided to decision-makers	Expand the permanent, non-partisan research staff workforce serving the legislature (e.g., California Research Bureau)
	Designate a third-party staffing agency to hire and employ legislative research staff, making them state employees rather than employees of political parties
	Designate third-party agency to select expert witnesses using objective criteria
	Develop guidelines for research evidence summaries provided to committee members (e.g., prohibit policy recommendations, require grading of evidence, employ rigorous peer review prior to distribution)
	Require transparency from decision-makers, including documentation of the research evidence used to support policy decisions

^*^ https://uccs.ucdavis.edu/policy-experts-catalog and https://www.calstatela.edu/univ/ppa/journalist/guide.php

#### Recommendation 1: Increase Capacity to Engage with and Utilize Non-partisan Research Evidence

Findings suggest that legislators and their staff face severe time constraints that limit their ability to utilize non-partisan research evidence in health policymaking, which diminishes the quality of legislation and erodes public trust in government.^28^ Various approaches to increase capacity to engage with non-partisan research evidence include adopting bill introduction limits, which 14 states currently use;^29^ creating a more balanced bill-to-staff ratio; implementing fees for bill introductions; revising deadline systems to evenly distribute workload across legislative sessions; revising legislative calendars to incorporate mechanisms for flexibility; and adopting “aging requirements” that allot committee members a specific amount of time to review legislation before voting. A total of 27 states currently use aging requirements, but timeframes vary widely.^30^ For instance, while California permits committees to vote immediately after bill printing, New Jersey mandates a 6-day interval between printing and voting.^30^

#### Recommendation 2: Invest in Formal University–Government Partnerships

Findings suggest that investing in university-government partnerships can promote non-partisan research evidence use in health policymaking.^31^ States have been increasingly investing in science policy fellowship programs,^32, 33^ which facilitate relationship-building between researchers and policymakers and commission policy-focused research with the rigor and impartiality of the scientific process. Incentivizing academics to conduct policy-focused research with special grants, recognition, and funded positions could further promote research evidence use in the policymaking process.^34^

#### Recommendation 3. Bolster the Credibility of Research Evidence Provided to Decision-makers

Findings demonstrate that legislators feel more confident about the credibility of research evidence and their ability to utilize it with support from non-partisan knowledge broker agencies such as CHBRP.^35–37^ While these agencies come with a cost, states can invest in them via recurring budgetary funds, public–private partnerships, and taxes on consumer goods harmful to public health.^38, 39^ States can further bolster the credibility of research evidence by mitigating the effects of partisanship on research presentation in committees, including by delegating legislative research staff hiring practices to non-partisan agencies and implementing mechanisms that restrict political leadership from stifling debate and manipulating votes. This could include stipulating that all members receive equal funding for their offices regardless of party affiliation or voting history.

### Limitations

Interviews were conducted within a single state, which may limit generalizability. Furthermore, most interviews were conducted prior to the COVID-19 pandemic; two additional interviews were completed in 2021, which were consistent with our previous findings. In addition, distinctions between individual- and institutional-level barriers may be somewhat arbitrary and dependent on scale; however, our literature review supplemented our data and verified our coding schema. Finally, we did not include the perspectives of academicians. Findings suggest that analogous institutional reforms within academia should be explored.

## CONCLUSION

Although conducting research studies at the pace of policymaking may not be realistic, we have identified institutional barriers that contribute to the evidence-policy gap and institutional solutions supported by participants that can aid state legislators in making evidence-based decisions that influence the health and safety of our communities. Findings suggest that the creation and expansion of non-partisan knowledge broker agencies, especially those that are university-affiliated, is a worthwhile goal. Doing so may bridge the “know-do gap,” thereby improving health outcomes through evidence-based policymaking.

### Supplementary Information

Below is the link to the electronic supplementary material.

Supplementary file1 (DOCX 58 KB)

## Data Availability

All data generated or analyzed during this study are included in this published article and its supplementary information files.
