# Supplementary material for: Perspectives of California Legislators on Institutional Barriers and Facilitators to Non-Partisan Research Evidence Use in State Health Policymaking
Source: J Gen Intern Med. 2023 Dec 15;39(9):1704–12. doi: 10.1007/s11606-023-08547-z (PMC11255140; doi:10.1007/s11606-023-08547-z)
Supplement: Supplementary file 1 — Supplementary file1 (DOCX 58 KB) [file 11606_2023_8547_MOESM1_ESM.docx]

**APPENDICES**

| **Appendix A. Glossary of Terms** | |
| --- | --- |
| **Term** | **Definition** |
| California Research Bureau (CRB) | A nonpartisan research agency that provides objective research and analysis to California state legislature staff, and the Governor's office. The CRB was established in 1953 by the California Legislature and is located within the California State Library. |
| California Health Benefits Review Program (CHBRP) | An independent research organization funded by state health plans that provides independent analysis of medical, financial and public health impacts of all proposed health insurance benefit mandates and repeals to the California state legislature. |
| Policy committee | A group of legislators responsible for reviewing and analyzing proposed legislation in a specific policy area. Policy committees are responsible for holding hearings on bills and other measures within their jurisdiction, where they hear testimony from stakeholders, experts, and members of the public. Committee members may also ask questions of witnesses, debate the merits of the proposals under consideration, and ultimately make recommendations to the full Legislature on whether to pass, amend, or reject the proposed legislation. They may also hold informational hearings to examine emerging policy issues at the chair’s discretion. |
| Committee chair | Most senior member of a committee who exercises significant authority over their legislative process. Chairs’ powers include:   1. Setting the committee’s agenda: Chairs have the authority to decide which bills will be considered and set deadlines for introducing new bills or submitting amendments. 2. Presiding over meetings: Chairs lead meetings, call on members to speak, and rule on procedural questions. 3. Appointing subcommittees, convening special sessions, and selecting expert witnesses. 4. Managing the committee’s staff: Chairs hire and supervise staff members including legislative analysts, consultants, and administrative staff. 5. Making voting recommendations to committee members and the full Legislature: Chairs have the authority to recommend whether bills should be passed, amended or rejected. |
| Committee co-chair | Member of a committee who shares the chair's responsibilities. The co-chair often assists with setting the committee's agenda, presiding over meetings, and making decisions on the committee's legislative partnerships. In some cases, the co-chair may take on additional responsibilities, such as leading subcommittees or serving as the committee's spokesperson. |
| Committee hearing | A committee meeting convened for the purpose of gathering information on a subject (i.e. informational hearing) or considering specific legislative measures (i.e. policy hearing). |
| Knowledge brokers | Individuals or entities who bridge evidence producers (i.e., researchers) to evidence users (i.e., policymakers) |
| Legislative office staff | Legislative office employees responsible for staffing legislation and preparing background materials, talking points, and hearing and floor statements for legislators. Includes legislative directors and legislative aides. |
| Legislative research staff | Legislative researchers who are appointed and employed by committee leadership (i.e. chair and co-chair) and whose primary responsibility is to provide objective and impartial advice on policy issues within their jurisdiction via bill analyses prior to committee hearings, as well as employees of state agencies and departments responsible for providing the legislature with research evidence to inform policymaking. |

**Appendix B. Interview Guide**

Part 1: Background experience

- Please tell me a bit about your work and any relevant experience.
- *If not legislative office, please answer the following questions:*
  - Agency type: non-profit, for-profit, think tank, professional organization, university, research network
  - Agency role: conducting research, synthesizing research, disseminating research
  - Resources (employees, funding, technology, training, etc.)
  - Partisan or nonpartisan

Part 2: Current flow of evidence to policymakers

1. We realize that policymakers must weigh several factors during decision-making, with health sciences research evidence being only one of them. In light of other factors (e.g., values, fiscal considerations, government agenda, political risk), how heavily is health sciences research weighed?
2. How does health sciences research information flow to state policymakers?
   1. For what purpose?
   2. When policymakers need information about an issue, how do they decide what type of information to request? (e.g. health sciences research evidence vs. economic data)
   3. How do policymakers decide whether health sciences research evidence is credible, important, and actionable?
   4. Is there a mechanism for policymakers to communicate health sciences research needs to researchers?
3. How does your organization/office work to bridge health sciences research evidence and policy? To what extent is it successful? Best practices at your organization or others? Other ideas?

Part 3: Solutions

1. We’ve seen the following solutions proposed or recommended. Could you please give us your thoughts on each?

| **Intervention** | **Examples** |
| --- | --- |
| *Research push*- researcher efforts to communicate research to policymakers more effectively | |
| Train researchers to design and disseminate research for uptake and use by policymakers | Knowledge translation toolkits and/or in-person training sessions |
| Incentivize researchers to conduct policy-relevant research and disseminate results | Reward via promotions or increased research funding |
| *Policymaker pull*- policymaker efforts to engage with research evidence more effectively | |
| Training policymakers to access and utilize research evidence | Ontario Ministry of Health offers 1-day seminar for policymakers |
| Maintaining a policymaker-targeted website that provides optimally packaged, topical reviews | PPD/CNCC database contains >800 systematic policymaker-targeted reviews |
| Laws mandating the examination of research in policy and program development (in the form of tools, templates, or guidelines) | Mexico’s 2004 Social Development Law requires new social development policies and interventions to be formally monitored and evaluated |
| *Exchange*- efforts to facilitate interaction/dialogue between researchers and policymakers | |
| Programs linking university researchers to policymakers | Wisconsin Family Impact Seminars (initiative of the Robert M. La Follette School of Public Affairs) presents policymakers with research evidence relevant to current legislation |
|  | Committee Connect is a program that elicits policymaker research needs and connects them to academic experts in relevant fields |
| Legislature-established research bodies | Washington State Institute for Public Policy (WSIPP)- researches public policy issues of interest to the legislature and state agencies, in association with The Evergreen State College |
| Government-mandated working groups | UK’s NICE mandates hearings at which pre-circulated evidence summaries are the starting point for deliberations between experts from various backgrounds, policymakers, and other stakeholders |
| Provision of honorary appointments for academics within public health bodies and vice-versa | NHS Scotland appoints Honorary Consultants who are academic experts from various fields to provide health information to the NHS and Scottish government |
| *Knowledge brokering*- efforts to synthesize evidence for policymaker use | |
| Government-affiliated institutions acting as knowledge brokers | CA Research Bureau compiles research information in response to state policymaker requests |
|  | UK’s NICE has an official mandate to provide or synthesize evidence for policy |
| Non-government institutions acting as knowledge brokers | Think tanks (e.g., Pew) |
|  | Research networks (e.g., Cochrane US Network & Cochrane library) |
|  | Professional organizations (e.g., AAP, CMA) |
|  | Academic journals (e.g., Health Affairs) |
|  | University health policy centers |

1. What organizational changes within universities and/or governments would encourage the use of health sciences research evidence by policymakers?
2. How might these changes be enacted in California? What barriers do you perceive?
3. Should legislation support and guide the production and use of health sciences research evidence for health policy? Other approaches? Explain.

Part 4:

1. Participant demographics: gender, race/ethnicity, age (<25 years, 26-39 years, >= 40 years)
2. Are there any individuals and/or stakeholder groups we should speak with? If so, whom?
3. Do you give us permission to share your name in a list of participants? We would keep your identity separate from your responses.

**Thank you!**
